# Future Scenarios for Levodopa-Induced Dyskinesias in Parkinson’s Disease

**DOI:** 10.3389/fneur.2015.00076

**Published:** 2015-04-01

**Authors:** Antonio Cerasa, Giacomo Koch, Alfonso Fasano, Francesca Morgante

**Affiliations:** ^1^IBFM, National Research Council, Catanzaro, Italy; ^2^Laboratorio di Neurologia Clinica e Comportamentale, Fondazione Santa Lucia IRCCS, Rome, Italy; ^3^Morton and Gloria Shulman Movement Disorders Clinic and the Edmond J. Safra Program in Parkinson’s Disease, Division of Neurology, Toronto Western Hospital, UHN, University of Toronto, Toronto, ON, Canada; ^4^Dipartimento di Medicina Clinica e Sperimentale, Università di Messina, Messina, Italy

**Keywords:** levodopa-induced-dyskinesias, hyperkinetic motor disorders, neuroimaging, neurobiology, neuropsychology, striato-cortical pathways

After its first use in clinic since 1960, oral administration of l-3,4-dihydroxyphenylalanine (levodopa) remains the main treatment for Parkinson’s disease (PD) patients. Although the vast majority respond positively to treatment, a significant proportion of PD patients develop daily fluctuations in mobility and troublesome involuntary movements known as levodopa-induced dyskinesias (LIDs). The time-to-onset and severity of this motor complication show large individual variability thus limiting the long-term use of levodopa and clinical strategies aimed at reducing LIDs manifestation. In the last few years, a considerable effort has been made to understand the neurobiological basis of this motor complication. In particular, recent evidence coming from human and animal studies has strongly contributed to reduce the gaps in our knowledge of LIDs pathogenesis.

The papers in this research topic highlight several themes relevant to the understanding of the clinical and neurobiological basis of LIDs. From a neuropsychological perspective, Pietracupa et al. (1) have explored the clinical correlated of the poor awareness of LIDs in PD patients; since the few studies conducted so far have used different methods and different patient populations, several hypotheses have been postulated, which suggest that several possible mechanisms may be implicated. One of the most recently proposed mechanisms emphasizes the role of proprioceptive and sensorimotor deficits associated with the impairment of the posterior parietal-ponto-cerebellar pathways, as nicely tested in a sample of PD patients with and without LIDs in the study by Stevenson et al. (2). Furthermore, using a top-down approach, the present topic firstly presents the latest neurophysiological findings in human models obtained using neuroimaging techniques and then moves toward cellular and molecular mechanisms of LIDs in animal model.

Finlay et al. (3) systematically adopted this approach in reviewing the recent literature with the scope to define a translation strategy that can bridge animal studies (that allow to assess the precise effects of drug treatment) with neuroimaging data on humans. These authors also discussed the last evidence provided by our works (4, 5), where we demonstrated that LIDs patients are characterized by anatomical and functional abnormalities of the prefrontal cortex involving the supplementary motor area (SMA) and the inferior frontal cortex (IFC). This notion has been recently confirmed by two independent groups investigating LIDs patients during the ON phase of levodopa therapy (6, 7). In particular, Herz et al. (6) investigated brain functional activity of LIDs during a motor task, continuously for 45 min after levodopa intake before the beginning of peak-dose dyskinesias. They found that PD patients with dyskinesias display an immediate hypersensitivity of the SMA and putamen to levodopa. Moreover, abnormal resting-state functional connectivity between the IFC and the putamen was demonstrated in PD patients with LID at 60 min after levodopa intake, consistent with the expected time peaks of dyskinesia (7).

Beyond the multiple phenomenology of LIDs in PD patients, further insights on the pathophysiology of hyperkinetic movement disorders derive from studies conducted in patients with idiopathic dystonia, tardive dyskinesia (TD), and Gilles de la Tourette syndrome (GTS). Dystonia is a hyperkinetic movement disorder characterized by sustained or intermittent muscle contractions causing abnormal, often repetitive, movements, postures, or both (8). The term TD referred to abnormal movements produced by the chronic exposure to dopamine receptor blocking agents. GTS is a childhood-onset complex neurobehavioral disorder defined by motor and phonic tics, which can be often complicated by comorbid conditions that could progress to behavioral changes. Recent neuroimaging papers demonstrate the presence of anatomical abnormalities involving the IFC in all these disorders (9–11). That is, we have hypothesized a common pathophysiological mechanism for all these hyperkinetic disorders, including LIDs, based on the inability by specific prefrontal areas to suppress involuntary movements (12, 13). Accordingly, other papers in this topic addressed this hypothesis (14, 15). Overviewing PET-related findings, Alongi et al. (14) discussed how this neuroimaging technique has demonstrated, both in idiopathic dystonia and GTS, metabolic and neurotransmission abnormalities not limited to the cortico-striato-pallido-thalamo-cortical pathway and also within the cerebello-thalamo-cortical network. Tessitore et al. (15) highlight a similar application for fMRI technique in PD patients, mainly discussing the usefulness of resting-state connectivity analysis. Blood oxygen level-dependent functional MRI signal recorded while the subject lies at rest with eyes closed represents an important tool for understanding brain disorders in patients with PD. Brain regions with similar functions have been shown to display robust functional connectivity during rest, which reflects the presence of direct and indirect anatomical pathways. For this reason, these authors proposed that in the near future, the practical application of this technique might provide a reliable MRI biomarker for an early diagnosis of PD.

Involvement of cortical areas (motor areas and prefrontal cortices) as well of the cerebellum in patients with LIDs has been also suggested by transcranial magnetic stimulation (TMS) studies, specifically using protocols to probe synaptic plasticity in humans (7, 16, 17). In the current research topic, Kishore et al. (18) propose a pathophysiological model of LIDs in PD in which aberrant impaired plasticity in the motor cortex might be sustained by deficient cerebellar modulation of sensory afferents to the motor cortex itself. In this comprehensive review, the authors outline functional and anatomical studies on basal ganglia circuits and their reciprocal connection with the cerebellum, as well the role of dopamine on the basal ganglia, the cortical motor areas and the cerebellum. The model proposed by Kishore et al. (18) allows to understand why non-invasive cerebellar stimulation may be efficacious in treating LIDs in PD (17). Notably, this notion parallels the aforementioned involvement of cortico-cerebellar pathways in the pathological processes of predictive motor control in LIDs (2).

The role of cortical areas such the IFC and the primary motor cortex and well of the cerebellum in the generation of LIDs is a novel concept supported by several recent studies reviewed in this research topic. Nevertheless, the cortico-striato-pallido-thalamo-cortical loop represents a critical network for the generation of LIDs as supported by the long-term beneficial effect of ablative surgery and deep brain stimulation (DBS) of the globus pallidus internus (GPi) and the subthalamic nucleus (STN) (19). Accordingly, one of the reviews of the present research topic is focused on the role of stereotactic surgery as a powerful tool to treat LIDs in PD (20). From ablative techniques (pallidotomy and subthalamotomy) to DBS of the GPi and the STN, the authors discuss the evidence for a direct anti-dyskinetic effect by techniques involving the GPi or the anteromedial portion of the STN, likely by current spreading to the pallido-thalamic bundle.

The current research topic is also enriched by three intriguing reviews related to the experimental models of LIDs. Animal models provide the unique opportunity to investigate in depth the molecular and cellular machinery involved in the pathophysiology of LIDs. Cenci (21) provides a comprehensive overview of the pre-synaptic mechanisms of LIDs, focusing on the central notion that the breakdown of pre-synaptic dopaminergic homeostasis predisposes to large fluctuations in therapeutic levels of dopamine upon treatment with levodopa (21). She also underlines the relevance of molecular, physiological, and morphological changes at post-synapatic level produced by dopaminergic denervation (21). One of the main concepts addressed in this review is the role of serotonin neurons in pathophysiological mechanisms of LIDs. Indeed, during the past few years, an abundant literature has support the hypothesis, although quite controversial, that LIDs may depend upon dopamine release from serotonin neurons. This hypothesis is the also the main subject of the review by Carta and Tronci (22). These authors revised the experimental evidence pointing to the role of serotonin neurons in producing dyskinesia, also discussing the clinical implications. Indeed, over the course of PD, other cellular compartments can substitute the lost dopaminergic neurons in mediating levodopa conversion to dopamine. In this context, the serotonergic system has emerged, in recent years, as a key player. In comparison to dopaminergic neurons, serotonin neurons share the same enzymatic machinery required to convert levodopa to dopamine and to store dopamine after exogenous administration of levodopa. However, serotonin neurons lack a feedback control mechanism able to fine-tune the synaptic levels of dopamine. Carta and Tronci (22) propose that this form of dopamine is released in an uncontrolled manner, leading to excessive synaptic dopaminergic peaks, and contributing to swings in synaptic dopaminergic levels following oral administration of levodopa. Although the authors discuss the vast body of evidence providing support for a major role of the serotonergic system in the appearance of LIDs in animal model, they also point out how clinical evidence for a role of serotonin modulation in attenuating LIDs in PD patients are still scarce and generally disappointing.

With a different perspective, Morin and Di Paolo report relevant studies investigating glutamate receptor subtypes in relation to motor complications in PD patients and 1-methyl-4-phenyl-1,2,3,6-tetrahydropyridine (MPTP)-lesioned monkeys (23). MPTP-lesioned primates are very useful to test potential antidyskinetic and/or anti-parkinsonian pharmacological agents. Glutamate receptors are reported to interact with numerous neurotransmitters and neuromodulators implicated in the development of LIDs including dopaminergic neurotransmission. The authors put the accent on the evidence that nigrostriatal denervation in PD leads to increased glutamatergic transmission in the basal ganglia and that glutamate receptor stimulation is involved in the pathogenesis of levodopa-induced motor complications in PD and glutamate receptor subtypes, such as mGlu5 and NMDA receptors, are potential selective targets for treatment of these adverse effects.

In conclusion, the contributions included in this research topic highlight the role of different cortical and subcortical areas as well as of other neurotransmitters beyond dopamine in the pathophysiology of LIDs. We hope that this research will stimulate new thinking about neurobiological mechanisms of LIDs.

## Conflict of Interest Statement

The authors declare that the research was conducted in the absence of any commercial or financial relationships that could be construed as a potential conflict of interest.
